# A Robust Dynamic Classifier Selection Approach for Hyperspectral Images with Imprecise Label Information

**DOI:** 10.3390/s20185262

**Published:** 2020-09-15

**Authors:** Meizhu Li, Shaoguang Huang, Jasper De Bock, Gert de Cooman, Aleksandra Pižurica

**Affiliations:** 1 GAIM, Department of Telecommunications and Information Processing, Ghent University, 9000 Gent, Belgium; aleksandra.pizurica@ugent.be; 2 FLip, Department of Electronics and Information Systems, Ghent University, 9052 Gent, Belgium; jasper.debock@ugent.be (J.D.B.); gert.decooman@ugent.be (G.d.C.)

**Keywords:** robust classification, dynamic classifier selection, hyperspectral images, noisy labels, imprecise probabilities

## Abstract

Supervised hyperspectral image (HSI) classification relies on accurate label information. However, it is not always possible to collect perfectly accurate labels for training samples. This motivates the development of classifiers that are sufficiently robust to some reasonable amounts of errors in data labels. Despite the growing importance of this aspect, it has not been sufficiently studied in the literature yet. In this paper, we analyze the effect of erroneous sample labels on probability distributions of the principal components of HSIs, and provide in this way a statistical analysis of the resulting uncertainty in classifiers. Building on the theory of imprecise probabilities, we develop a novel robust dynamic classifier selection (R-DCS) model for data classification with erroneous labels. Particularly, spectral and spatial features are extracted from HSIs to construct two individual classifiers for the dynamic selection, respectively. The proposed R-DCS model is based on the robustness of the classifiers’ predictions: the extent to which a classifier can be altered without changing its prediction. We provide three possible selection strategies for the proposed model with different computational complexities and apply them on three benchmark data sets. Experimental results demonstrate that the proposed model outperforms the individual classifiers it selects from and is more robust to errors in labels compared to widely adopted approaches.

## 1. Introduction

Hyperspectral images (HSIs) provide detailed spectral information about the imaged objects in hundreds of narrow bands, thereby allowing differentiation between materials that are often visually indistinguishable. This has led to numerous applications of hyperspectral imaging in various domains, including geosciences [1,2], agriculture [3,4,5], defense and security [6,7] and environment monitoring [8,9,10].

Supervised classification plays a vitally important role for analyzing HSIs by assigning image pixels into distinct categories or classes of interest available in a scene, based on a relatively small amount of annotated examples. Recent comprehensive surveys on HSI classification in remote sensing include [11,12,13,14,15]. It is generally agreed upon that incorporating spatial context together with spectral information leads to better classification results than using spectral information alone [16,17,18,19,20]. Further improvements in the classification accuracy can be obtained by combining multiple data sources, e.g., by augmenting HSI data with Light Detection and Ranging (LiDAR) data [21,22,23], Synthetic Aperture Radar (SAR) data [24,25] and/or high-resolution colour images [26,27,28]. Fusion of these multiple data sources is typically accomplished at feature level [29,30,31], or at decision level [26,32,33]. The concept of multiple classifier systems has been widely studied as a method for designing high performance classification systems at the decision level [34,35,36]. Among these, the Dynamic Classifier Selection (DCS) [37,38] approach selects the classifier that yields the highest probability of being classified correctly. By design, the combined classifier outperforms all the classifiers it is based on [33,39,40]. The key idea of DCS is to identify the best classifier dynamically for each sample from a set of classifiers. This classifier is usually selected based on a local region of the feature space where the query sample is located in. Most works use the K-Nearest Neighbors technique (grouping samples with similar features) to define this local region [41,42,43]. Then, for a given unseen sample the best classifier is estimated based on some selection criteria [44,45]. In this work, we group samples differently, by incorporating the robustness concept to the model specification.

While current machine learning systems have shown excellent performance in various applications [46,47,48], they are not yet sufficiently robust to various perturbations in the data and to model errors to make them reliably support high-stakes applications [49,50,51]. Therefore, increasing attention is being devoted to various robustness aspects of the models and inference procedures [52,53,54]. The work in [55,56] proposed robust methods by adopting empirical Bayesian learning strategies to parameterize the prior and used this Bayesian perspective for learning autoregressive graphical models and Kronecker graphical models. Earlier work by one of us [57] analyzed the global sensitivity of a maximum a posteriori (MAP) configuration of a discrete probabilistic graphical model (PGM) with respect to perturbations of its parameters, and provided an algorithm for evaluating the robustness of the MAP configuration with respect to those perturbations. For a family of PGMs, obtained by perturbation, the critical perturbation threshold was defined as the maximum perturbation level that does not alter the MAP solution. In classification problems, these thresholds determine the level to which the classifier parameters can be altered without changing its prediction. The experiments in [57] empirically showed that instances with higher perturbation thresholds tend to have a higher chance of being classified correctly (when evaluated on instances with similar perturbation thresholds). We combined this property with DCS and applied it to classification in our earlier work [58], but only as a proof of concept for toy cases with binary classes and two classifiers. In a follow up work [59] we presented an abstract concept of how the robustness measures can be employed to improve the classification performance of DCS in HSI classification.

Here we develop a novel robust DCS (R-DCS) model in a general setting with multiple classes and multiple classifiers, and use it to take into account the imprecision of the model that is caused by errors in the sample labels. The main novelty lies in interpreting erroneous labels as model imprecision and addressing this problem from the point of view of the robustness of PGMs to model perturbations; this also sets this work apart from our previous—more theoretical—work on robustness of PGMs [57,58,59], which did not consider the problem of erroneous label. The main issue with erroneous labels, also referred to as noisy labels [60,61], is that they mislead the model training and severely decrease the classification performance [62,63,64]. Recent works that address this problem usually focus on noisy label detection and cleansing [65,66,67]. However, detection of erroneous labels is never entirely reliable, and their correction even less so, especially when the sample labels are relatively scarce or spatially scattered across the image. Thus, it is imperative to study the robustness of classifier models under different levels of label noise and to understand how the performance of different classifiers deteriorates with label noise.

We hypothesize that the framework of imprecise probabilities can offer a viable approach to improve a classifier’s robustness to low-to-moderate amounts of label noise. Therefore, we build our robust DCS (R-DCS) model based on an imprecise probabilistic extension of a classical PGM. Particularly, we build on Naive Bayes Classifiers (NBCs), but it is possible to extend the proposed framework to other classification models. We use an adapted version of the Imprecise Dirichlet Model (IDM) [68] to perturb the local probability mass functions in the model to corresponding probability sets. This imprecise probabilistic extension of an NBC is called a Naive Credal Classifier (NCC) [69]. The amount of perturbation of such an NCC is determined by a hyperparameter that specifies the degree of imprecision of the IDM. The maximum value of the hyperparameter under which the NCC still remains determinate—yields a single prediction—is the perturbation threshold of the NCC. Such perturbation thresholds essentially show how much we can vary the local models of the NBC without changing the prediction result, thereby providing us with a framework for dealing with model uncertainty.

The influence of label noise on the classification performance was studied earlier from a different perspective in [70,71]. The work in [70] showed experimentally that NBCs yield favourable performance in the presence of label noise compared to classifiers based on KNN, support vector machine (SVM) and decision trees [72]. These conclusions were based on empirical classification results on thirteen synthetic datasets, designed for several selected problems from the UCI repository [73]. The work in [71] empirically analyzed the effect of class noise on supervised learning on eight medical datasets. Only the end classification results were analysed in both works.

Here we take a different approach and we characterise statistically the effect of erroneous labels on statistical distributions of features and on the estimated spectral signatures of landcover classes. The empirical results explain from this perspective clearly the reasons for the considerable robustness of NBCs to label noise and at the same time they also show how erroneous labels affect the actual conditional distributions of features given the class labels, introducing inaccuracies in the classification process. This motivates us to employ the framework of imprecise probabilities to develop a classifier that is more robust to model uncertainties. As a first step, we use this framework to analyze the effect of noisy labels on the robustness of the predictions of NBCs.

Next, we put forward our robust DCS (R-DCS) model and first apply it to HSIs classification in the presence of noisy labels. We perform dynamic selection among two classifiers: one based on spectral and the other based on spatial features. Both of these are formulated as an NBC. Specifically, we apply a principal component analysis (PCA) to extract spectral features from HSIs, where the decorrelating property of PCA justifies the conditional independence assumption that NBC relies on. The spatial features are generated by applying morphological operators on the first five principal components (PCs). We also apply our R-DCS model to a multi-source data set that includes HSI and LiDAR data. For this data set, we perform dynamic selection among three classifiers: the two classifiers corresponding to HSI and a third classifier based on the elevation information in the LiDAR data.

For the selection criteria for our R-DCS, we define three selection strategies—R-T, R-LA and R-EU—that differ in computational complexity. R-T simply selects the classifier with the highest perturbation threshold. While computationally efficient, this approach does not always perform well because the exact relation between perturbation thresholds and performance differs from one classifier to another. Two other strategies are proposed to improve upon this by determining empirical relations between the perturbation thresholds of different classifiers and their probabilities of correctly classifying the considered instance. Particularly, the empirical probabilities of correctly classifying the test sample are estimated based on the training samples that are closest to the test sample in terms of a given perturbation distance. In R-LA, the perturbation distance between two data samples is defined by the absolute value of the difference in their perturbation thresholds for a given classifier. R-EU defines this distance as the Euclidean distance in a space spanned by the perturbation thresholds of all the considered classifiers. R-EU is computationally more complex but outperforms the others in most cases of practical importance. Experimental results on three real data sets demonstrate the efficacy of the proposed model for HSIs classification in the presence of noisy labels. In the two HSI data sets, the R-EU strategy performs best among the three selection strategies when the label noise is relatively low, while R-T and R-LA offer better performance when the label noise is rather high. In the multi-source data set, the R-EU strategy always outperforms the other methods in all cases.

The main contributions of the paper can be summarized as follows:

(1) We characterise statistically the effect of label noise on the estimated spectral signatures of different classes in HSIs and on the resulting probability distributions (both prior probabilities and conditional probabilities given the class label). This analysis provides insights into the robustness of NBC models to erroneous labels, and at the same time it shows in which way errors in data labels introduce uncertainty in the involved statistical distributions of the classifier features.
(2) We further analyze the effect of noisy labels on the robustness of NBCs and, in particular, on perturbation thresholds of the corresponding NCCs. The results show that this robustness decreases as the amount of label noise that is applied increases.
(3) In order to cope with this decrease in robustness due to label noise, we propose a robust DCS model, dubbed R-DCS, which selects classifiers that are more robust, using different selection strategies that are based on the critical perturbation thresholds of the involved classifiers. In particular, we provide three possible selection strategies: R-T, R-LA and R-EU. Two of these (R-T and R-LA) already appeared in a preliminary version of this work [59], but in a more abstract set-up, without exploring their performance in the presence of label noise. The third selection strategy, R-EU, proposed here, is computationally more complex, but performs better than the other two in cases with low to moderate levels of label noise (up to 30%), which are of most interest in practice.
(4) The proposed R-DCS models are validated on three real data sets. Compared to our conference paper [59], we take into account the label noise into HSI classification and conduct more experiments to evaluate the proposed model. The results reveal that the proposed model outperforms each of the individual classifiers it selects from and is more robust to errors in labels compared to some common methods using SVM and graph-based feature fusion.

The rest of this paper is organized as follows. Section 2 contains preliminaries, reviewing briefly the basic concept behind NBC, its imprecise-probabilistic extension NCC and the notion of perturbation thresholds that we build upon. Section 3 describes three representative (hyperspectral and hybrid) real data sets that we use in our experiments. The following two sections contain the main contributions of this work: Section 4 is devoted to the statistical characterisation of the influence of noisy labels on spectral signatures, on statistical distributions of the classifier features and on perturbation thresholds. In Section 5, the proposed model R-DCS is described and three possible selection strategies based on imprecise-probabilistic measures are defined. The overall proposed framework for robust dynamic classifier selection for hyperspectral images is presented and discussed. Experimental results on the three real data sets are reported in Section 6. The results demonstrate that the proposed model outperforms each classifier it selects from. Comparing to the competing approaches such as SVM and graph-based feature fusion, the proposed model proves to be more robust to label errors, inheriting this robustness from the NBCs that are at its core. A detailed discussion of the main results and findings is presented in Section 7, and Section 8 concludes the work.

## 2. Preliminaries

We first introduce the basic concept behind Naive Bayes Classifiers (NBCs) in this section. Next, imprecise extensions of NBCs—called Naive Credal Classifiers (NCCs)—are introduced and their perturbation thresholds are defined.

### 2.1. Naive Bayes Classifiers

Let *C* denote the class variable taking values *c* in a finite set C. We denote by $F_{i}$ the *i*-th feature variable taking values $f_{i}$ in a finite set $F_{i}$, i∈{1,⋯,m}, where *m* is the number of features. For notational convenience, we gather all feature variables in a single vector $F=(F_{1},\cdots ,F_{m})$ that takes values $f=(f_{1},\cdots ,f_{m})$ in $F_{1}\times \cdots \times F_{m}$.

For any given feature vector f, an NBC returns the Maximum a Posteriori (MAP) estimate of the class variable *C*, assuming the conditional independence $P\left(f|c\right)=\prod_{i=1}^{m}P(f_{i}|c)$. The estimated class is thus:   

(1) $$\begin{array}{cc} \; & \hat{c}=\arg\max_{c\in C}P\left(c|f\right)=\arg\max_{c\in C}\frac{P\left(c\right)\prod_{i=1}^{m}P(f_{i}|c)}{\sum_{c^{\prime }\in C}P\left(c^{\prime }\right)\prod_{i=1}^{m}P(f_{i}|c^{\prime })}. \end{array}$$

The involved (conditional) probabilities are typically estimated from data. To avoid falsely estimated zero probabilities due to empirical estimation, we adopt the common method of Laplace smoothing [74,75,76], meaning that for all i∈{1,...,m}, c∈C and $f_{i}\in F_{i}$:

(2) $$\begin{array}{c} P\left(c\right):=\frac{n(c)+1}{n+|C|},\;P\left(f_{i}|c\right):=\frac{n(c,f_{i})+1}{n\left(c\right)+|F_{i}|},\;\;\;\;\;\;\; \end{array}$$

where *n* is the total number of data points, n(c) is the number of data points with class *c* and $n(c,f_{i})$ is the number of data points with class *c* and *i*-th feature $f_{i}$.

### 2.2. Naive Credal Classifiers and Perturbation Thresholds

The Naive Credal Classifier (NCC) [69] is an extension of the Naive Bayes Classifier to the framework of imprecise probabilities that can be used to robustify the inferences of an NBC. Basically, the idea is to consider an NBC whose local probabilities are only partially specified.

In particular, instead of considering a probability mass function P(C) that contains the probabilities P(c) of each of the classes c∈C, an NCC considers a set of such probability mass functions, which we denote by P(C). Similarly, for every class c∈C and every i∈{1,⋯,m}, it considers a set $P(F_{i}|c)$ of conditional probability mass functions. In general, these local sets can be learned from data, elicited from experts, or obtained by considering neighbourhoods around the local models of an NBC. We here consider the first option. In particular, we use a version of the Imprecise Dirichlet Model (IDM) [68], suitably adapted such that it is guaranteed to contain the result of Laplace smoothing. In particular, P(C) is taken to belong to P(C) if and only if there is a probability mass function *t* on C such that

$$P\left(c\right)=\frac{n(c)+1+st(c)}{n+|C|+s}\;\mathrm{for}\;\mathrm{all}\;c\in C,$$

where *s* is a fixed hyperparameter that determines the degree of imprecision. For every i∈{1,⋯,m} and c∈C, the local set $P(F_{i}|c)$ is defined similarly.

If we now choose a single probability mass function P(C) in P(C) and, for every c∈C and i∈{1,⋯,m}, a single conditional probability mass function $P(F_{i}|c)$ in $P(F_{i}|c)$, we obtain a single NBC. By doing this in every possible way, we obtain a set of NBCs. This set is a Naive Credal Classifier (NCC) [69].

Classification for such an NCC is done by performing classification with each of the NBCs it consists of separately. If all these NBCs agree on which class to return, then the output of the NCC will be that class. If they do not agree, the result of the NCC is indeterminate and consists of a set of possible classes, amongst which it is unable to choose.

The maximum value of *s* for which the result of the NCC is still determinate is a particular case of the critical perturbation threshold defined in [57]. It provides a numerical indication of the robustness of the NBC’s prediction with respect to changes to the probabilities that make up the model. Furthermore, it has also been observed that for any given instance, the corresponding critical perturbation threshold serves as a good indicator for the performance of the original NBC: instances with higher thresholds are classified correctly more often [57]. In the following, we denote this perturbation threshold by $s^{\left(\mathrm{per}\right)}$ and we compute it from the data at hand using the algorithm in [57].

## 3. Datasets

We conduct our experiments on two real HSI datasets: *Salinas Scene* and *HYDICE Urban*, and a multi-source data set *GRSS2013*.

**Figure 1:**
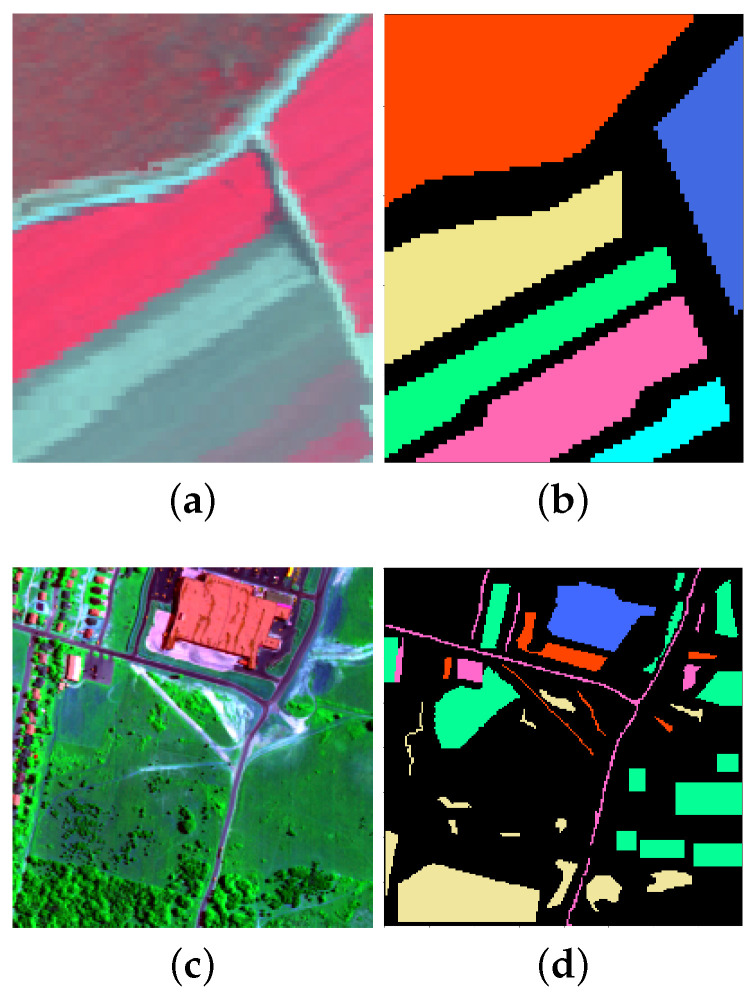
Two real hyperspectral data sets used in the experiments. (**a**) False color images of the selected part of *Salinas Scene* and (**b**) the corresponding ground truth classification. (**c**) False color image of the selected part of *HYDICE Urban* and (**d**) the corresponding ground truth classification.

The *Salinas Scene* dataset was gathered by the AVIRIS sensor with 224 bands in 1998 over Salinas Valley, California. The original data set consists of 512×217 pixels with a spatial resolution of 3.7 m per pixel. It includes 16 classes in total. For our experiments, we select a typical region of size 100×80 shown with a false color image in Figure 1a. There are six classes in this region, as listed in Table 1, which also shows the number of labeled samples per class. Figure 1b shows the ground truth spatial distribution of these classes.

The *HYDICE Urban* dataset was captured by the HYDICE sensor. The original data contains 307×307 pixels, each of which corresponds to a 2×2 m${}^{2}$ area. There are 210 wavelengths ranging from 400 nm to 2500 nm, resulting in a spectral resolution of 10 nm. In our experiments, we use a part of this image with size 200×200 shown in Figure 1c. The number of bands was reduced from 210 to 188 by removing the bands 104–108, 139–151 and 207–210, which were seriously polluted by the atmosphere and water absorption. Detailed information on the available classes and the number of samples per class is given in Table 2. The ground truth classification is shown in Figure 1d.

**Figure 2:**
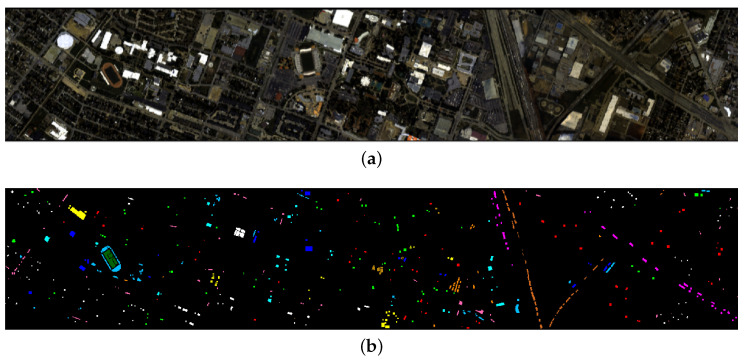
The False color images (**a**) and the corresponding ground truth classification (**b**) of the data set *GRSS2013*.

Our third data set, *GRSS2013*, was a benchmark data set for the 2013 IEEE GRSS data fusion contest [77]. This data set, consisting of HSI and LiDAR data was acquired over the University of Houston campus and the neighboring urban area in June 2012. The HSI has 144 spectral bands and 349 × 1905 pixels, containing in total 15 classes as shown in Table 3. The false color image is shown in Figure 2a and the ground truth classification is shown in Figure 2b.

## 4. Robustness Analysis with Noisy Labels

We now move on to analyze the effect of errors in labels on the estimation of spectral signatures of landcover classes and on the estimated statistical distributions of the classes and of features given the class labels. This analysis gives an insight into how errors in labels introduce uncertainty about the models that various classifiers rely upon. In order to study (and further on improve) the robustness to these model uncertainties, we adopt the framework of imprecise probabilities.

### 4.1. Model Uncertainties Due to Noisy Labels

Here we analyze the influence of label noise on the estimated spectral signatures of different classes in an image as well as the effect on the estimated prior probabilities of the given classes and the conditional probability distributions of the features given the class label. The experiments here are conducted with NBCs. The NCC framework, which was introduced in Section 2.2, will be used further on to define the perturbation thresholds that will be employed in our new model.

**Figure 3:**
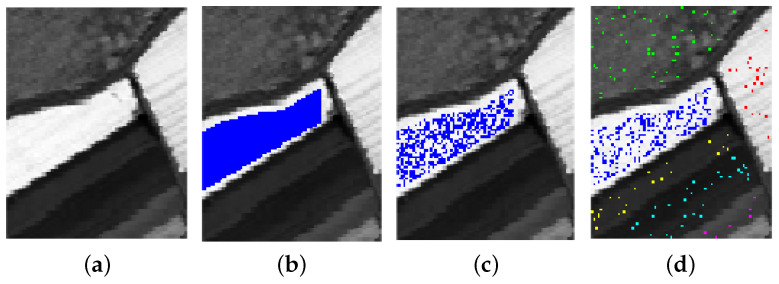
An illustration of introducing label noise. (**a**) the first PC of *Salinas Scene*; (**b**) labelled samples in Class 1 (marked in blue); (**c**) training samples (50% of the labelled samples) in Class 1 and (**d**) an instance of the samples labelled as Class 1 when ρ=0.5. Different colors denote samples from different classes that were erroneously flipped to Class 1.

We conduct experiments on the two real HSIs described in Section 3 and shown in Figure 1. To reduce the data dimensionality, PCA is commonly applied on the original HSIs data. We use discretized principal component values as the features for NBCs. Due to the decorrelating property of PCA, these features are conditionally independent given the class label, and thus conform to the assumption of NBC. In all the experiments, the PC values are uniformly discretized into twenty intervals. We define the level of label noise ρ as the proportion of training samples that have wrong labels. These erroneous labels are chosen at random in C∖{c} as in [63], with C the set of class values and *c* the true class. Figure 3 shows an illustration of introducing label noise in the *Salinas Scene* dataset. The first PC is shown in Figure 3a. All the labelled samples in Class 1 are highlighted in Figure 3b. Next, we randomly select 50% of the highlighted samples as the training samples for Class 1 (Figure 3c). We also select at random a given portion ρ of the total training samples (from various classes) and flip each of them to one of the remaining classes at random. Figure 3d illustrates an instance of the resulting Class 1 labels for ρ=0.5. Different colours denote different original classes of the training samples that were flipped to Class 1. Note that the choice of ρ is here merely for clearer illustration purposes; a situation with 50% of wrong labels is unlikely to be relevant in practice.

**Figure 4:**
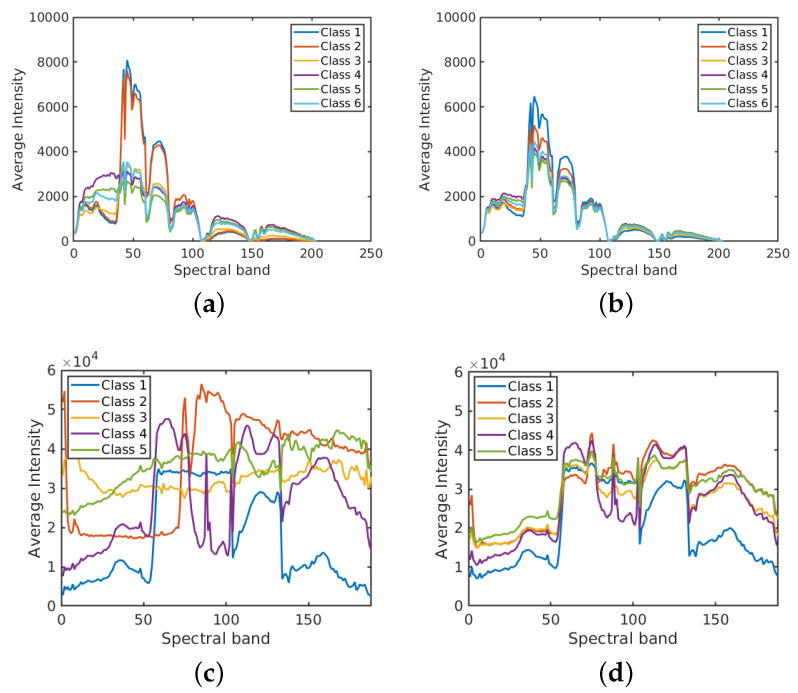
Average spectral signatures for *Salinas Scene* and *HYDICE Urban*. (**a**) *Salinas Scene* with ρ=0; (**b**) *Salinas Scene* with ρ=0.5; (**c**) *HYDICE Urban* with ρ=0; (**d**) *HYDICE Urban* with ρ=0.5.

Figure 4 shows the average spectral signatures of each class on two real HSI data sets: *Salinas Scene* and *HYDICE Urban*. These average spectral signatures are obtained by taking the mean value of spectral intensities for each class. Without label noise the spectral signatures of different classes show different trends. In the presence of label noise, the spectral signatures falsely appear to be more similar to each other, which will affect inevitably the classification accuracy. Observe that when ρ=0 the spectral responses of Class 1 and Class 2 in *Salinas Scene* are quite similar. This is because the materials corresponding to theses two classes are similar (two types of brocoli). For *HYDICE Urban*, the spectral signatures without label noise are rather different from each other and the effect of erroneous labels is evident. In both cases, label noise obviously tends to uniformise all the spectral signatures as expected, because now each of them is computed from a mixture of different classes.

**Figure 5:**
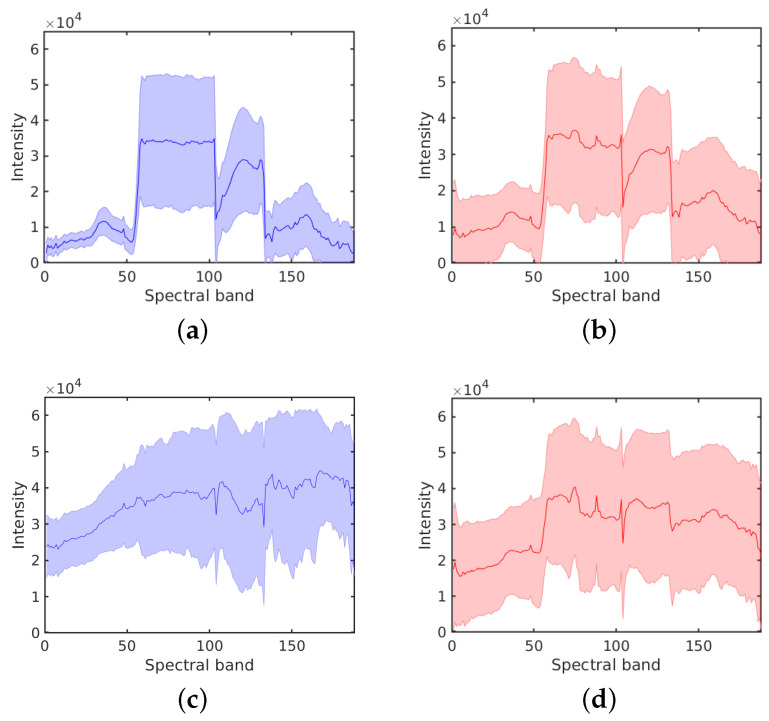
Estimated spectral signatures from the labeled data for two classes from *HYDICE Urban*, without label noise (left) and with label noise ρ=0.5 (right). The solid line shows the average spectral signature and the shaded region denotes the standard deviation. (**a**) Class 1 with ρ=0; (**b**) Class 1 with ρ=0.5; (**c**) Class 5 with ρ=0; (**d**) Class 5 with ρ=0.5.

**Figure 6:**
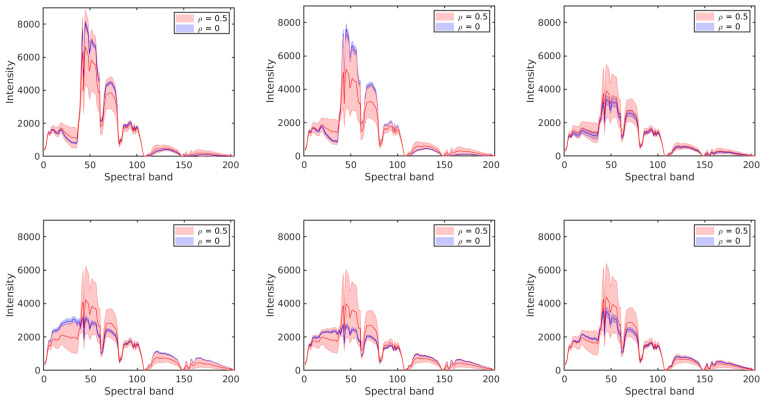
Spectral signatures for Class 1–6 (from top left to bottom right) with different levels of label noise for the *Salinas Scene* data set.

Figure 5 shows average spectral signatures together with their standard deviation regions, for two different classes from *HYDICE Urban*, without label noise and with label noise ρ=0.5. The solid line in the middle shows for a given class the mean value of the pixel intensities in each spectral band, whereas the shaded region denotes the standard deviation over all the labeled pixels in that class. With erroneous labels, the spread gets larger and the means of the intensities change as well. Figure 6 shows overlays of spectral signatures for ρ=0 and ρ=0.5 for the six classes from another test image (*Salinas Scene*). We observe again that errors in labels result in wider shaded regions (larger standard deviations) and in decreased differences between the average spectral signatures of different classes due to the mixing effect, echoing the results in Figure 4.

**Figure 7:**
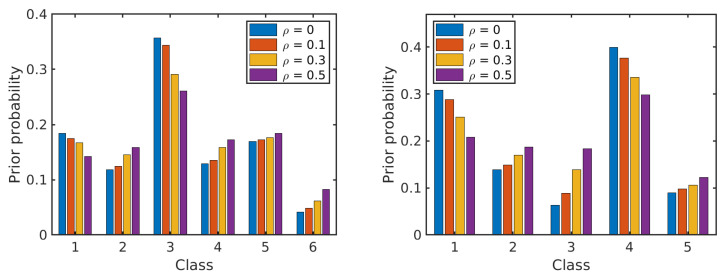
The effect of erroneous labels on prior probabilities of classes in the two data sets: *Salinas Scene* (**left**) and *HYDICE Urban* (**right**).

**Figure 8:**
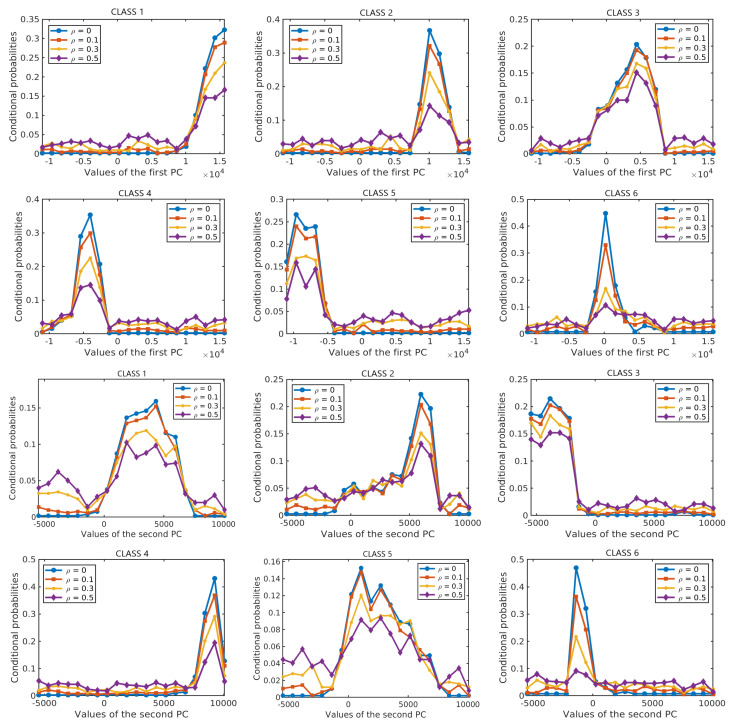
Conditional probabilities of the first PC (**top six diagrams**) and the second PC (**bottom six diagrams**) for different levels of label noise in the *Salinas Scene* data set.

**Figure 9:**
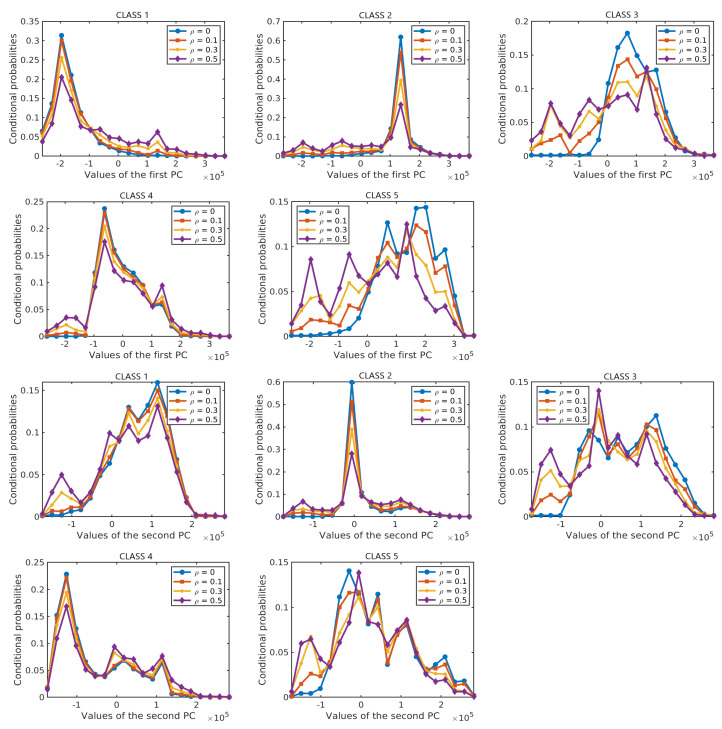
Conditional probabilities of the first PC (**top five diagrams**) and the second PC (**bottom five diagrams**) for different levels of label noise in the *HYDICE Urban* data set.

Next, we analyze the effect of label noise on the estimated prior probabilities of different classes and the conditional probability distributions of the features given the class label. The prior probabilities and the conditional probabilities are computed by Equation (2). Each of the results is obtained as an average over 10 runs on different training samples. In each run, 50% of the labelled samples from each class are selected at random as training samples and their labels are perturbed at random according to the given ρ. Figure 7 shows the effect of label noise on prior probabilities of classes with ρ∈{0,0.1,0.3,0.5} in the two data sets. While the actual prior probabilities of different classes are significantly different from each other, e.g., for the *Salinas Scene* data set, p(C=3)=0.36 and p(C=6)=0.04 for ρ=0, these differences become smaller when label noise increases. Figure 8 and Figure 9 show the probability distributions of the first two PCs in the two data sets, respectively, given the class label and with different levels of label noise ρ∈{0,0.1,0.3,0.5}. For the *Salinas Scene* data set, when ρ=0.5, the distribution conditioned on class 6 changed a lot in both PCs, since there are relatively few labelled samples from class 6 in this data set. The distributions conditioned on other classes keep a similar shape when increasing ρ from 0 to 0.5, but the peak values decrease and the distribution shape gets more and more flat compared to the distributions without label noise. The distributions of the second PC show similar behaviour, becoming more flat when the level of label noise increases. The conditional distributions for the *HYDICE Urban* data set show similar behavior to those for the *Salinas Scene* data set.

The presented results provide insights into how erroneous labels affect the estimation of the spectral signatures per class as well as the prior probabilities of the classes and the distributions of the classifier features (i.e., the underlying statistical model that governs the operation of NBCs and other related statistical models) conditioned on the class label. The analysis of these results demonstrates clearly that erroneous labels lead to model uncertainties, which will in their turn affect the classification performance. In order to mitigate this, models that are robust to model uncertainty are needed. We build such a robust classifier using the framework of imprecise probabilities. In particular, we adopt NCCs, an extension of NBCs, which allows us to assess how robust an NBC is with respect to model uncertainty. A clever dynamic selection among multiple NBCs will then lead to a robust dynamic classifier selection approach that we advocate in this work.

### 4.2. Impact of Noisy Labels on Perturbation Thresholds

An NCC provides an elegant way to account for model uncertainties by extending the probability mass functions in an NBC to corresponding sets of probabilities. Recall that the perturbation threshold of an NCC $s^{\left(\mathrm{per}\right)}$ is defined as the maximum value of *s* under which the NCC remains determinate. Here we analyze the effect of noisy labels on these perturbation thresholds. We conduct experiments on the *Salinas Scene* data set.

**Figure 10:**
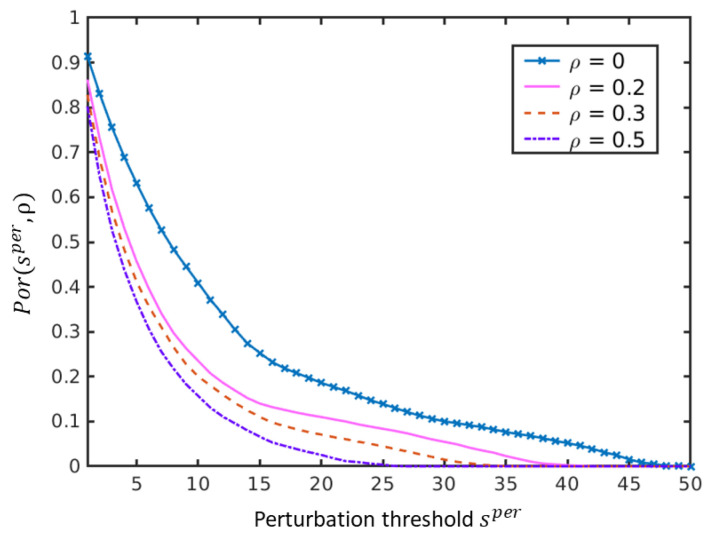
The effect of erroneous labels on perturbation thresholds of NCCs estimated empirically on the *Salinas Scene* data set. The portion of samples whose perturbation threshold is above a given value $s^{\left(per\right)}$ is plotted for different values of ρ and denoted by $Por(s^{\left(per\right)};\rho )$. Observe that this portion $Por(s^{\left(per\right)};\rho )$ drops when ρ is larger indicating that with increasing the label noise the performance becomes less reliable on more and more samples.

Figure 10 shows the correlation between the level of label noise and the perturbation thresholds. Let $\mathrm{Por}(s^{\left(\mathrm{per}\right)};\rho )$ denote the portion of samples whose perturbation thresholds are larger than $s^{\left(\mathrm{per}\right)}$ under the label noise level ρ. The family of curves $\mathrm{Por}(s^{\left(\mathrm{per}\right)};\rho )$ in Figure 10 shows clearly that the portion of samples whose perturbation thresholds are above a given level drops when the level of label noise increases. In other words, the perturbation thresholds for some samples get smaller when the level of label noise increases. This means that, as expected, the robustness of the classification will decrease when ρ is larger. Given the correlation between robustness and accuracy [57], this will result in a lower accuracy as well. In order to mitigate this drop in robustness (and hence accuracy), we will now develop a method that uses perturbation thresholds to minimize this unwanted effect.

## 5. Robust Dynamic Classifier Selection (R-DCS)

In this section, we develop a robust dynamic classifier selection (R-DCS) model to improve the classification performance under noisy labels. We first introduce some notation and then propose three possible selection strategies to estimate the best classifier among a set of available ones. Finally, an application of these R-DCS models in hyperspectral image classification is presented.

### 5.1. Notation

Let $\Psi =\{\psi_{1},\psi_{2},...,\psi_{L}\}$ be a pool of base classifiers that are used for DCS. In particular, each $\psi_{l}\in \Psi$ is an NBC. Let $X=\{x_{i}\}$ be a set of training samples and $Y=\{y_{i}\}$ a set of testing samples. The methods described below can be applied in a general context, but in our hyperspectral image application, the samples $x_{i}\in R^{m}$ and $y_{i}\in R^{m}$ are vectors composed of pixel values at a particular spatial location in *m* spectral bands. We denote by $s_{l,i}^{\left(\mathrm{per}\right)}$ the perturbation threshold of the *l*-th classifier ($\psi_{l}$) in sample *i*.

### 5.2. Selection Strategies for R-DCS

The key idea of a DCS is to try and find the classifier with the highest probability of being correct for a given unseen sample. We propose a classifier selection approach making use of the observation that, for a given fixed classifier, instances with higher perturbation thresholds tend to have a higher chance of being classified correctly. Based on this general concept, we provide three concrete selection strategies employing perturbation thresholds as follows.

#### 5.2.1. The R-T Strategy

In order to select the most competent classifier among a set of available ones, a first idea is simply to choose the classifier with the highest perturbation threshold for each sample. We refer to this strategy as R-T.

Let $\lambda_{j}\in \left\{1,...,L\right\}$ denote the index of the base classifier that will be assigned to sample *j*. The R-T strategy selects for each test sample $y_{j}$ the classifier $\psi_{\lambda_{j}}\in \Psi$ that exhibits the highest perturbation threshold:

(3) $$\begin{array}{c} \lambda_{j}=\arg\max_{l\in \{1,...,L\}}s_{l,j}^{\left(per\right)} \end{array}$$

#### 5.2.2. The R-LA Strategy

Instead of analysing each sample individually, we now take into account a local surrounding region of the image sample. This local surrounding includes the nearest neighbors of the test sample in terms of a given distance. First we define this distance metric for each classifier separately and we refer to this strategy as R-LA. In particular, for each classifier we choose *N* training samples whose perturbation thresholds are closest to that of the test sample. To that end, we define the perturbation distance $d_{l}\left(x_{i},x_{j}\right)$ between two data samples $x_{i}$ and $x_{j}$ as the absolute value of the difference between their perturbation thresholds for the classifier *l*:

(4) $$\begin{array}{c} d_{l}\left(x_{i},x_{j}\right)=\left|s_{l,i}^{\left(per\right)}-s_{l,j}^{\left(per\right)}\right|. \end{array}$$

Furthermore, we let $N_{l,j}$ be the set of *N* training samples that are the nearest neighbors of $y_{j}$ in terms of $d_{l}\left(x_{i},y_{j}\right)$. For each sample $y_{j}$ to be classified, we then determine the most competent classifier $\psi_{\lambda_{j}}$ as follows:

(5) $$\begin{array}{cc} \; & \lambda_{j}=\arg\max_{l\in \{1,...,L\}}\frac{|{\tilde{N}}_{l,j}|}{|N_{l,j}|}=\arg\max_{l\in \{1,...,L\}}\frac{|{\tilde{N}}_{l,j}|}{N}, \end{array}$$

where ${\tilde{N}}_{l,j}$ is the subset of $N_{l,j}$ composed of those training samples that are correctly classified by $\psi_{l}$.

**Figure 11:**
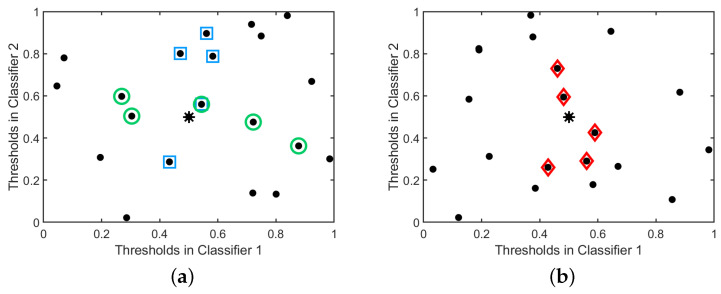
An illustration of the R-LA and R-EU strategies for the case with two classifiers. The star denotes the test sample and we set N=5. (**a**) R-LA forms two sets of nearest neighbours: one based on $d_{1}$ (for Classifier 1, denoted by squares) and the other based on $d_{2}$ (for Classifier 2, denoted by circles). The classifier yielding the largest number of correctly classified samples from the corresponding set, is selected. (**b**) R-EU defines one common set of nearest neighbours for both classifiers (based on $d_{eu}$, denoted by diamonds). The classifier that correctly classifies the largest number of samples in this common set is now selected. (**a**) R-LA strategy; (**b**) R-EU strategy.

Figure 11a illustrates this strategy with an artificial example with twenty training instances and two classifiers.

#### 5.2.3. The R-EU Strategy

The R-EU strategy also aims to choose a classifier based on a local surrounding of the image sample in terms of the perturbation thresholds, but now a common set of nearest neighbors is defined based on a single perturbation distance. We define this common perturbation distance as the Euclidean distance in the space spanned by the perturbation thresholds of the different classifiers:

(6) $$\begin{array}{c} d_{eu}\left(x_{i},x_{j}\right)=\sqrt{{\left(s_{1,i}^{\left(per\right)}-s_{1,j}^{\left(per\right)}\right)}^{2}+{\left(s_{2,i}^{\left(per\right)}-s_{2,i}^{\left(per\right)}\right)}^{2}+...+{\left(s_{L,i}^{\left(per\right)}-s_{L,i}^{\left(per\right)}\right)}^{2}}, \end{array}$$

where $s_{k,i}^{\left(per\right)}$ and $s_{k,j}^{\left(per\right)}$ are the threshold values of the *k*-th classifier for the sample $x_{i}$ and $x_{j}$, respectively and *L* is the total number of classifiers. Now let $N_{eu,j}$ be the set of *N* training samples that are the nearest neighbors of $y_{j}$ in terms of $d_{eu}\left(x_{i},y_{j}\right)$. For each sample $y_{j}$ to be classified, we then estimate the most competent classifier $\psi_{\lambda_{j}}$ as follows:

(7) $$\begin{array}{cc} \; & \lambda_{j}=\arg\max_{l\in \{1,...,L\}}\frac{|{\tilde{N}}_{eu,l,j}|}{|N_{eu,j}|}=\arg\max_{l\in \{1,...,L\}}\frac{|{\tilde{N}}_{l,j}|}{N}, \end{array}$$

where ${\tilde{N}}_{eu,l,j}$ is the subset of $N_{eu,j}$ composed of those training samples that are correctly classified by $\psi_{l}$.

Figure 11b illustrates this strategy with a fictitious example involving twenty training samples and two classifiers.

### 5.3. Discussion of the Proposed Strategies

Among the three proposed selection strategies for R-DCS model, the R-T strategy is the simplest one. It operates on each sample separately, selecting the best classifier according to their perturbation thresholds for the particular sample. However, it ignores the fact that the exact relation between perturbation thresholds and performance may differ from one classifier to another. The other two strategies R-LA and R-EU improve upon this by incorporating information from a local surrounding of the test sample in the perturbation thresholds space. R-LA, which is based on absolute distance between the samples, is computationally simpler than R-EU, which is based on Euclidean distances. Notably, R-T is a parameter-free method, and for R-LA and R-EU only a simple parameter *N* needs to be chosen, which is particularly convenient from a practical point of view.

### 5.4. R-DCS in Hyperspectral Image Classification

**Figure 12:**
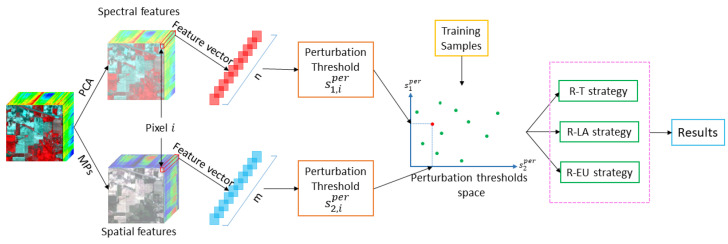
The proposed method for hyperspectral image classification based on Robust Dynamic Classifier Selection (R-DCS).

We apply the proposed R-DCS model in hyperspectral image classification. The proposed model can be seen as an ensemble learning method, which consists of classifiers with different input features. We conduct experiments in the subsequent sections on three real remote sensing data sets, including two HSIs and one multimodal data set (HSI+LiDAR). In the experiments with HSIs alone, we employ the spectral and spatial features of HSIs as the inputs of our method as illustrated in Figure 12. While in the experiment with HSI and LiDAR, apart from the two features of HSIs, an additional feature with altitude information of objects from LiDAR is utilized. In Figure 12, we construct two classifiers, one operating on spectral and the other one on spatial features. PCA is employed for spectral feature extraction and morphological profiles [78] for spatial feature extraction. A morphological profile is constructed by the repeated use of morphological openings and closings with a structuring element of increasing size. In this work, we extract spatial features by morphological profiles composed of morphological openings and closings with partial reconstruction on PCs, similarly as in [78,79].

Note that in the general case, the R-DCS framework allows us to assign an arbitrary number of feature vectors to every pixel and feed each of those feature vectors to its own classifier. In the particular scheme from Figure 12, however, we assign to every pixel in a HSI two feature vectors: one composed of spectral features and the other composed of spatial features. For each of these feature vectors, the corresponding perturbation threshold is calculated, as explained in Section 2.2, using the algorithm of [57]. A dynamic classifier selection is then conducted for each test sample according to one of the selection strategies from Section 5.2. In the case of more than two types of features from one or more data sources, we need to calculate the perturbation thresholds for L≥2 feature vectors following the same procedure as above and apply the same selection strategies, which are already formulated in general for an arbitrary number *L* classifiers. Algorithm 1 shows the entire process of our R-DCS model for the case with *L* classifiers
**Algorithm 1** Robust dynamic classifier selection (R-DCS) model**Input**: Training samples X and corresponding labels $C_{train}$, testing samples Y, *N*, *L***Output**: Classification map $C_{out}$**Classification**:**for**l=1→L**do**    Classify the labels $C_{\psi_{l}}$ of Y with classifier $\psi_{l}$;    **for** each sample *i* in X and Y **do**        Compute perturbation threshold $s_{l,i}^{per}$;    **end for****end for****Dynamic selection**:**for** each sample *j* in Y    **R-T:** $C_{out}\left[j\right]\leftarrow c_{\psi_{\lambda_{j}}}$ according to Equation (3);    **for** each sample *i* in X **do**        **R-LA:** Compute $d_{l}\left(x_{i},y_{j}\right)$;        **R-EU:** Compute $d_{eu}\left(x_{i},y_{j}\right)$;    **end for**    **R-LA:** $C_{out}\left[j\right]\leftarrow c_{\psi_{\lambda_{j}}}$ according to Equation (5);    **R-EU:** $C_{out}\left[j\right]\leftarrow c_{\psi_{\lambda_{j}}}$ according to Equation (7);**end for do**

## 6. Experimental Results in HSI Classification

We evaluate the performance of our methods on three real data sets: two HSI data sets (*Salinas scene* and *Urban area HYDICE*) and a multi-source data set (*GRSS2013*), which contains HSI and LiDAR data. The details of the three data sets were described in Section 3.

In the following experiments, we extract the first 50 PCs for the spectral features as a compromise between the performance and complexity for all the methods. The morphological profiles for spatial features are generated from the first 5 principal components (representing more than 99% of the cumulative variance) of the HSI data with 5 openings and closings by using disk-shaped SE (ranging from 2 to 10 with step size increment of 2). The morphological profiles for elevation features are generated from the LiDAR data with 25 openings and closings by using disk-shaped SE (ranging from 2 to 50 with step size increment of 2). The values of each PC and morphological profile are uniformly discretized into 10 intervals.

We compare the proposed R-DCS model under different selection strategies with the following schemes:

(1) NBC implemented with spectral features alone (NBC-Spe), with spatial features alone (NBC-Spa) and with elevation features alone (NBC-LiDAR).
(2) K-nearest neighbors (KNN) classifier with spectral features of HSIs. The number of neighbors is obtained by five-fold cross validation over the training samples.
(3) Support vector machine (SVM) classifier with polynomial kernel, applied on spectral features.
(4) Generalized graph-based fusion (GGF) method of [29], which makes use of all types of features.

Observe that GGF and the proposed R-DCS combine different types of features, while other methods use one or the other type of features. The only parameter of the proposed model is the number of neighbors *N* in the R-LA and R-EU methods. We estimate this parameter by five-fold cross validation over the training samples, as we do for the KNN method. Three widely used performance measures are used for quantitative assessment: overall accuracy (OA), average accuracy (AA) and the Kappa coefficient (κ). Overall accuracy is the ratio between correctly classified testing samples and the total number of testing samples. Average accuracy is obtained by first computing the accuracy for each class and then considering the average of these accuracies. The Kappa coefficient, finally, measures the level of agreement between the ground truth and the classification result of the classifier [80]: κ=1 corresponds to complete agreement and hence a perfect classifier, whereas κ=0 corresponds to a classifier that ignores the feature vector and classifies purely at random. Let $n_{i,j}$ be the number of testing samples in class *i* that are labeled as class *j* by the classifier. Then OA, AA and κ are computed as:

(8) $$OA:=\frac{1}{n_{t}}\sum_{i}n_{i,i},\;\;\;AA:=\frac{1}{n_{c}}\sum_{i}\frac{n_{i,i}}{n_{i,+}},\;\;\;\kappa :=\frac{OA-EA}{1-EA},$$

where $n_{t}:=\sum_{i}\sum_{j}n_{i,j}$ is the total number of testing samples, $n_{c}$ is the number of classes, $n_{i,+}:=\sum_{j}n_{i,j}$ is the number of testing samples in class *i*, EA = $\sum_{i}\left(n_{i,+}/n_{t}\right)\left(n_{+,i}/n_{t}\right)$ is the expected accuracy of a classifier that ignores the feature vector and $n_{+,i}:=\sum_{j}n_{j,i}$ is the number of testing samples that are classified in class *i*. In the following experiments, 10 percent of the labeled samples are used for training and the rest are for testing. The reported experimental results are averages over 10 runs with different training samples.

### 6.1. Experiments on the HYDICE Urban Data Set

The first experiment is conducted on the *HYDICE Urban* data set. The reference classes and their corresponding number of labeled training and testing samples are shown in Table 4. The false color image and the ground truth are shown in Figure 1a,b.

The classification results for the *HYDICE Urban* data set with different levels of label noise are listed in Table 5, where the best result is marked in bold. All the three proposed strategies outperform the basic classifiers they select from with different levels of label noise. The proposed R-DCS model with the R-EU strategy outperforms the others in most cases in this data set. When the level of label noise is 0, the GGF method performs the best and the two NBC models: NBC-Spe and NBC-Spa are inferior to all other methods. However, all the three proposed strategies improve the performance of NBC with about 3% improvement over NBC-Spe and 5% improvement over NBC-Spa for the R-EU strategy. With increasing levels of label noise, the accuracy of the GGF method decreases heavily from 94% to 64%, and similarly for SVM from 91% to 58%. Remarkably, all of our methods show only a slight decrease in OA which is within 5%. The more complex strategy R-EU outperforms the more simply ones R-T and R-LA, especially for the cases with less errors in labels. Table 5 also demonstrates that the proposed methods mostly outperform (and the R-EU strategy always outperforms) all the reference methods in terms of all the performance measures (OA, AA and κ) in all cases where label noise exists. Table 6 shows the classification accuracy per class for the *HYDICE Urban* data set with different classifiers. We compare ρ=0 and ρ=0.5 to study the change in accuracy for each class in the presence of label noise. The results show that the class-specific accuracies mostly drop significantly due to the effect of label noise. Class 2 is an exception: label noise there increases the accuracies for NBC-Spe, NBC-Spa, KNN, SVM and R-T. We can also see from Table 6 that when ρ=0, the GGF method yields the highest accuracy in each class. Our proposed methods R-LA and R-EU outperform NBC-Spe and NBC-Spa in Class 2 with about 20% improvement. When ρ=0.5, all the methods perform badly in Class 3 due to the limited number of training samples. Our proposed methods show competing accuracies in the first three classes and the R-LA method performs the best in Class 4 and Class 5.

**Figure 13:**
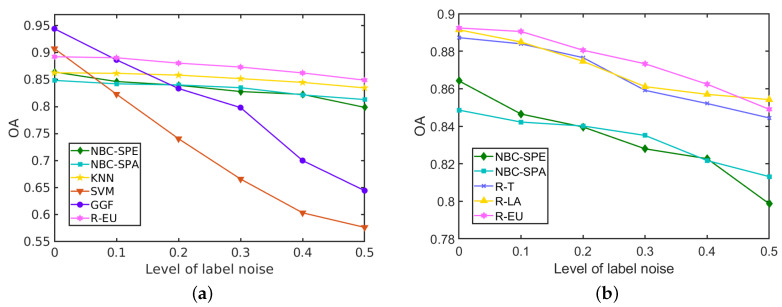
Influence of the label noise on the performance (in terms of OA of different methods) on the *HYDICE Urban* data set. (**a**) R-EU compared with other methods; (**b**) Comparisons among three strategies.

In Figure 13a we compare the performance of our best strategy for the *HYDICE Urban* data set, R-EU, with the reference methods. With increasing levels of label noise, the performance of the GGF and SVM deteriorates significantly, while NBCs, KNN and the proposed methods are more stable in terms of OA. The classification accuracy of all the three analysed strategies is depicted in Figure 13b for different levels of label noise. All the strategies outperform the individual NBCs (NBC-SPE and NBC-SPA) that they select from. When the level of label noise is less than 0.5, the R-EU method performs better, while the R-T and R-LA methods achieve higher accuracy at ρ=0.5. Compared to NBC with spatial features, accuracy improvement is above 3% at different levels of label noise, which is significant in the task of HSI classification. Compared to some of the most competitive methods SVM and GGF, an important improvement is obtained in the presence of label noise.

### 6.2. Experiments on the Salinas Scene Data Set

The second experiment is conducted on the *Salinas Scene* data set. Information about the classes and number of samples used for training and testing are listed in Table 7. The false color image and ground truth are shown in Figure 1.

**Figure 14:**
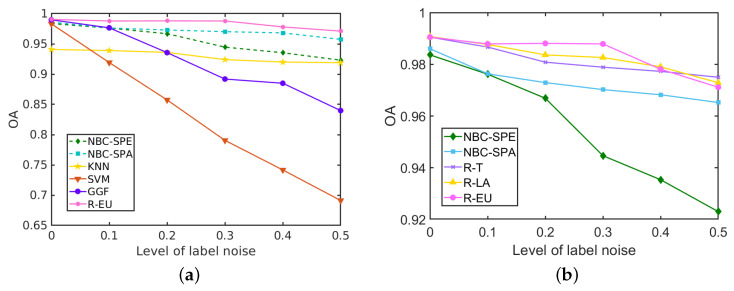
Influence of the label noise on the performance (in terms of OA of different methods) for the *Salinas Scene* data set. (**a**) R-EU compared with other methods; (**b**) Comparisons among three strategies.

The classification results for the *Salinas Scene* data set with different levels of label noise are depicted in Table 8 and Figure 14. The three strategies within the proposed method perform similarly due to the better performance of NBCs on this data set. The proposed R-DCS model under any of the three presented strategies outperforms all the other methods at every level of label noise, even when the level of label noise is 0. When the level of label noise increases, the accuracy of SVM drops heavily from 98% to 69%, and similarly for GGF the accuracy drops from 99% to 87%. The KNN’s accuracy drops only a little in this data set from 94% to 92%. For the proposed model, with any of the three selection strategies, the decrease in the accuracy is also only about 2%. It is interesting to notice that with lower levels of label noise, the R-EU strategy performs better than R-T and R-LA, while the opposite is true at larger levels of label noise.

### 6.3. Experiments on GRSS2013 Data Set

The third experiment is conducted on the *GRSS2013* data set. Information about the classes and number of samples used for training and testing are listed in Table 9. The false color image and ground truth are shown in Figure 2.

**Figure 15:**
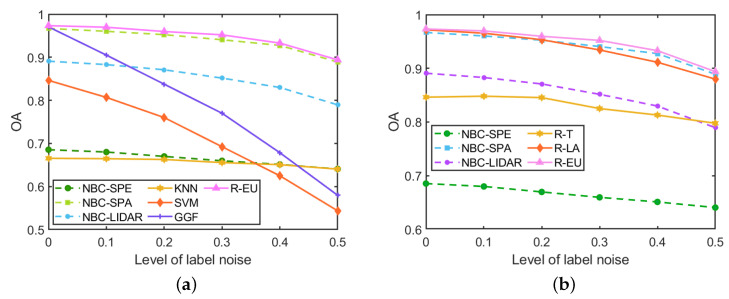
Influence of the label noise on the performance (in terms of OA of different methods) for the *GRSS2013* data set. (**a**) R-EU compared with other methods; (**b**) Comparisons among three strategies.

The classification results for the *GRSS2013* data set with different levels of label noise are depicted in Table 10 and Figure 15. Our proposed methods and the representative GGF method combine the spectral features, spatial features and elevation features in these experiments. The results show that our R-EU method yields the best performance in terms of OA, AA and κ at each level of label noise. R-LA outperforms the three NBCs, i.e., NBC-Spe, NBC-Spa and NBC-LiDAR, at low levels of label noise (ρ≤0.2), but performs worse than NBC-Spa when ρ>0.2. When label noise rises from 0 to 0.5, the OA of SVM drops heavily from 85% (ρ=0) to 54% (ρ=0.5) and similarly GGF has an OA decrease of 39%. KNN proves to be much more robust to the label noise—its OA decreases only by 3% in the same range of ρ values. Among our methods, R-T does not yield good results on this data set. Compared with R-LA, R-EU performs consistently better, demonstrating the effectiveness of the R-EU strategy.

### 6.4. Performance at Extremely Large Levels of Label Noise

The previous analysis showed that the performance of NBC-based classifiers and our approach that is build upon NBC remains remarkably stable even at large levels of label noise (ρ=0.5). To explain this behaviour and to explore at which levels of label noise this performance starts to drop, we also perform experiments with extremely large levels of label noise (ρ>0.5). For these experiments, we choose the selection strategy that yields the best performance the most times (R-T for the two HSI data sets and R-EU for the *GRSS2013* data set), and compare it with other methods.

**Figure 16:**
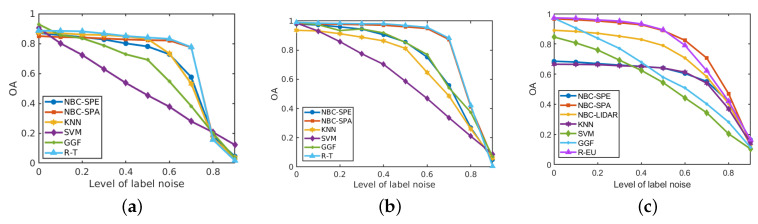
Influence of label noise on the performance (in terms of OA) of different methods on three data sets. (**a**) *HYDICE Urban*; (**b**) *Salinas Scene*; (**c**) *GRSS2013*.

**Figure 17:**
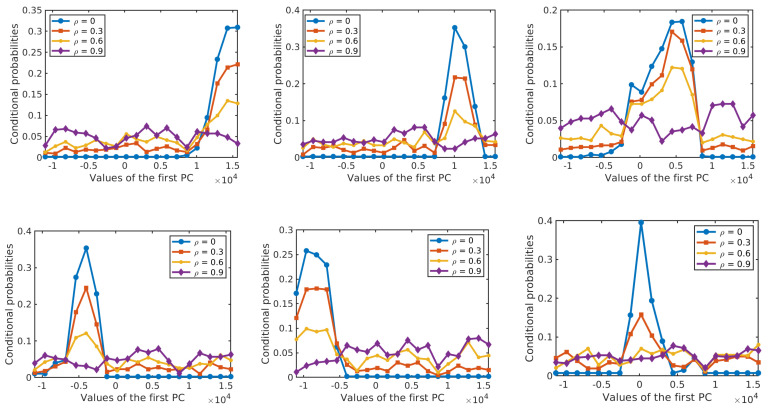
Conditional probabilities of the first PC for Class 1–6 (from the top left to bottom right) with different levels of label noise for the *Salinas Scene* data set.

**Figure 18:**
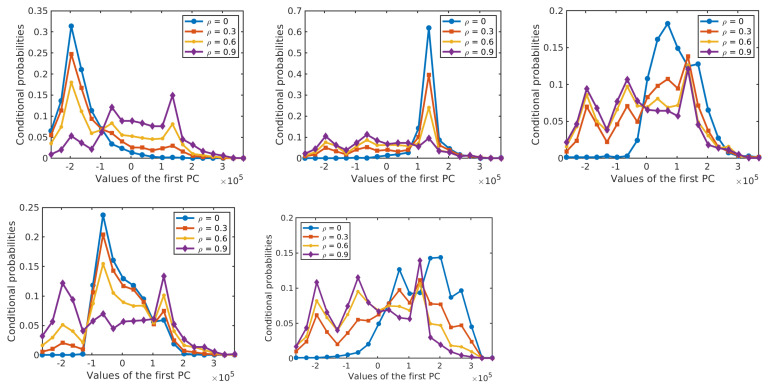
Conditional probabilities of the first PC for Class 1–5 (from the top left to bottom right) with different levels of label noise for the *HYDICE Urban* data set.

Figure 16 shows the performance of the resulting classifiers under different levels of label noise on the three analysed data sets. In the three data sets, the overall accuracy of NBC-Spe decreases gradually with increasing label noise in the range ρ<0.5, and it drops abruptly afterwards reaching a value near zero when ρ=0.9. The reason for this sharp decrease can be attributed to the flattening of the conditional densities for larger ρ as shown in Figure 17 and Figure 18 for the first PC in the two HSI data sets. (Note that the statistical distributions in Figure 17 and Figure 18 as well as the statistical distributions in Section 4 were obtained with 50% of the labeled samples per class in order to allow for a more reliable empirical estimation of the corresponding distributions.) NBC-Spa and our method R-T in the two HSI data sets show a similar evolution in both data sets and have a sudden drop in OA around ρ=0.7. KNN and GGF suffer from a significant performance drop at ρ=0.6, while SVM shows approximately linear decrease in both datasets. The trend exhibited by NBCs and our proposed method is much better than a linear decrease in the accuracy, because when the accuracy drops below a certain level the exact values do not matter anymore as all methods are useless in that range. In the *GRSS2013* data set, NBCs with different types of features and our method R-EU show a similar evolution and have a sudden drop in OA around ρ=0.7. KNN shows behaviour that is similar to NBC-Spe, while SVM and GGF almost decrease linearly in this data set.

## 7. Discussion

The experimental results presented in the previous section as well as the statistical characterization in Section 4, provide new insights into the effects of label noise on supervised classification of hyperspectral remote sensing images. Empirical conditional probability distributions of HSI features conditioned on class labels, as well as prior distributions of class labels, exhibited graceful flattening with increasing amounts of label noise. Their evolution clarifies why Bayesian classifiers such as the relatively simple NBCs are much more robust to label noise than some other, more complex methods. These results are consistent with previous findings from [70,71], where it was experimentally established that NBCs yield better classification accuracy in the presence of label noise compared to classifiers based on KNN, SVM, and decision trees.

Our experimental analysis shows also clearly that incorporating spatial features into the classification process not only improves the classification accuracy but increases robustness to label noise as well. It is well established that using spatial information typically improves the classification accuracy, and some recent works that addressed HSI classification in the presence of noisy labels [66,81] also incorporate spatial features.

We addressed the effect of label noise from the point of view of the robustness of probabilistic graphical models to model perturbations. We built a robust dynamic classifier selection method upon this reasoning. The proposed approach enjoys remarkable robustness to label noise, inherited from the naive Bayesian classifiers that lie at its core. We instantiated our general approach with three particular selection strategies that have different levels of complexity. The proposed approach improves upon the NBCs that it combines and lends itself to incorporating easily multiple data sources and multiple types of features. Both NBC and the proposed robust dynamic classifier exhibit a characteristic trend in the presence of label noise: the classification accuracy decreases very slowly until the level of label noise becomes excessively high (60% or more erroneous labels) and then it drops abruptly. The evolution of the probability distributions of HSI features and estimated priors for class labels provides a nice explanation for this behaviour as was pointed out in the previous section. Interestingly, classifiers based on SVM and on an advanced graph fusion method show a faster decrease of the classification accuracy with increasing levels of label noise. While these more sophisticated classifiers outperform the other analysed ones in the case of ideally correct labels, they appear to be rather more vulnerable to label noise and become inferior to NBCs, KNN and the proposed approach already when a small percentage of labels are erroneous.

Based on these results and findings, we believe that the following research directions are interesting to explore:

(1) Analyzing the performance of more advanced Bayesian classifiers, e.g., based on Markov Random Fields, in the presence of label noise.
(2) Exploring which levels of label noise are acceptable for a given tolerance in the classification accuracy and how robust are different learning models in this respect. This can significantly help in practice for optimizing the resources and ensuring the prescribed tolerance levels.
(3) Deep learning methods are becoming the dominant technology for supervised classification. It is well known already that these models tend to be extremely susceptible to various degradations in the data such as noise and to different adversarial attacks. It would be of interest to study thoroughly their behaviour in the presence of label noise. Motivated by the excellent performance of Bayesian models to erroneous labels, a natural idea to explore is how Bayesian approaches can be incorporated to improve the robustness of deep learning methods to label noise.

## 8. Conclusions

In this work, we started by analysing the effect of errors in data labels on the estimation of spectral signatures of landcover classes, on the estimated statistical distributions of features given the class labels and on the prior probabilities of the given classes. The analysis reveals that NBCs are remarkably robust to label noise but also that erroneous labels introduce uncertainties to models, which inevitably deteriorate the performance of all classifiers, including NBCs. To deal with the imprecision of the model that is caused by errors in the sample labels, we proposed a novel, robust dynamic classifier selection model, that we refer to as R-DCS. The R-DCS model is based on imprecise-probabilistic robustness measures and was applied to HSIs classification in the presence of errors in data labels. Three possible selection strategies are presented for the R-DCS based on the robustness measures. All the provided strategies outperform the classifiers they select from, but their performance differs for different levels of label noise. The R-EU strategy performs better than the other two in most cases, while R-T and R-LA enjoy the benefit of lower computational complexity than R-EU. The experimental results also demonstrate that the proposed model is more robust to label noise compared to some common classification approaches such as KNN, SVM and graph-based feature fusion.

## Figures and Tables

**Table 1:** Reference classes for the *Salinas Scene*.

No.	Class Name	Labelled Samples
1	Brocoli-green-weeds-1	1014
2	Brocoli-green-weeds-2	652
3	Grapes-untrained	1965
4	Lettuce-romaine-4wk	711
5	Lettuce-romaine-5wk	930
6	Lettuce-romaine-6wk	229
	Total	5501

**Table 2:** Reference classes for the *HYDICE Urban* data set.

No.	Class Name	Labelled Samples
1	Trees	3123
2	Concrete	1410
3	Soil	637
4	Grass	4044
5	Asphalt	912
	Total	10,126

**Table 3:** Reference classes for the *GRSS2013* data set.

No.	Class Name	Labelled Samples
1	Healthy grass	1251
2	Stressed grass	1254
3	Synthetic grass	697
4	Trees	1244
5	Soil	1242
6	Water	325
7	Residential	1268
8	Commercial	1244
9	Road	1252
10	Highway	1227
11	Railway	1235
12	Parking Lot 1	1233
13	Parking Lot 2	469
14	Tennis Court	428
15	Running Track	660
	Total	15,029

**Table 4:** Reference classes for the *HYDICE Urban* data set.

No.	Class Name	Train	Test	Labelled Samples
1	Trees	312	2811	3123
2	Concrete	141	1269	1410
3	Soil	64	573	637
4	Grass	404	3640	4044
5	Asphalt	91	821	912
	Total	1013	9113	10,126

**Table 5:** Classification results for the *HYDICE Urban* data set with different classifiers.

ρ	PM	NBC-Spe	NBC-Spa	KNN	SVM	GGF	R-T	R-LA	R-EU
0	OA	0.8642	0.8486	0.8627	0.9074	**0.9442**	0.8872	0.8914	0.8924
	AA	0.7904	0.8139	0.7556	0.8577	**0.8969**	0.8376	0.8299	0.8272
	κ	0.8090	0.7948	0.8075	0.8698	**0.9216**	0.8441	0.8484	0.8495
0.1	OA	0.8465	0.8422	0.8616	0.8230	0.8866	0.8839	0.8849	**0.8905**
	AA	0.7783	0.8019	0.7582	0.7751	0.8252	0.8319	**0.8336**	0.8297
	κ	0.7855	0.7869	0.8063	0.7530	0.8409	0.8400	**0.8536**	0.8477
0.2	OA	0.8396	0.8401	0.8583	0.7406	0.8336	0.8766	0.8746	**0.8805**
	AA	0.7592	0.7952	0.7531	0.6738	0.7599	**0.8222**	0.7987	0.8075
	κ	0.7754	0.7852	0.8039	0.6407	0.7630	0.8303	0.8247	**0.8332**
0.3	OA	0.8280	0.8351	0.8519	0.6660	0.7982	0.8591	0.8611	**0.8733**
	AA	0.7614	0.7672	0.7506	0.6195	0.7075	0.7952	0.7968	**0.8070**
	κ	0.7596	0.7766	0.8053	0.5450	0.7129	0.8068	0.8081	**0.8229**
0.4	OA	0.8227	0.8217	0.8449	0.6034	0.7002	0.8521	0.8570	**0.8624**
	AA	0.7375	0.7746	0.7488	0.5535	0.6435	0.7866	**0.7873**	0.7828
	κ	0.7513	0.7855	0.8080	0.4610	0.5175	0.8011	0.8050	**0.8083**
0.5	OA	0.7987	0.8131	0.8347	0.5765	0.6445	0.8444	**0.8542**	0.8490
	AA	0.7165	0.7012	0.7536	0.5201	0.6080	0.7504	**0.7805**	0.7627
	κ	0.7176	0.7467	0.8089	0.4292	0.5706	0.7871	**0.7956**	0.7890

**Table 6:** Classification accuracy per class for the *HYDICE Urban* data set with different classifiers.

ρ	Class	NBC-Spe	NBC-Spa	KNN	SVM	GGF	R-T	R-LA	R-EU
0	1	0.8908	0.9594	0.9584	0.9573	**0.9678**	0.9520	0.9466	0.9417
	2	0.7573	0.7959	0.7967	0.7904	**0.9883**	0.8454	0.9464	0.9756
	3	0.6841	0.7714	0.7592	0.7452	**0.8037**	0.7260	0.6672	0.7347
	4	0.9192	0.8066	0.8830	0.8967	**0.9538**	0.9135	0.9239	0.9343
	5	0.6102	0.8124	0.7418	0.7259	**0.7799**	0.7163	0.7372	0.7296
0.5	1	0.8406	0.9353	**0.9413**	0.8680	0.8538	0.9192	0.9100	0.8852
	2	0.7991	0.8779	**0.8810**	0.8180	0.8333	0.8582	0.8645	0.8264
	3	**0.6876**	0.3892	0.5044	0.6091	0.5298	0.5689	0.4101	0.4386
	4	0.7060	0.7637	0.7755	0.7071	0.7077	0.7681	**0.7772**	0.7712
	5	0.4278	0.5810	0.4566	0.3069	0.5068	0.4971	**0.7151**	0.6447

**Table 7:** Reference classes for the *Salinas Scene* data set.

No.	Class Name	Train	Test	Labelled Samples
1	Brocoli-green-weeds-1	101	913	1014
2	Brocoli-green-weeds-2	65	587	652
3	Grapes-untrained	197	1768	1965
4	Lettuce-romaine-4wk	71	640	711
5	Lettuce-romaine-5wk	93	837	930
6	Lettuce-romaine-6wk	23	206	229
	Total	550	4951	5501

**Table 8:** Classification results for the *Salinas Scene* data set with different classifiers.

ρ	PM	NBC-Spe	NBC-Spa	KNN	SVM	GGF	R-T	R-LA	R-EU
0	OA	0.9836	0.9860	0.9408	0.9832	0.9898	0.9905	**0.9907**	0.9905
	AA	0.9792	0.9865	0.8434	0.9794	0.9847	0.9898	0.9900	**0.9920**
	κ	0.9789	0.9807	0.9231	0.9784	0.9869	0.9878	**0.9881**	0.9878
0.1	OA	0.9762	0.9763	0.9390	0.9192	0.9766	0.9867	0.9877	**0.9879**
	AA	0.9716	0.9810	0.8528	0.9105	0.9683	0.9878	0.9874	**0.9882**
	κ	0.9789	0.9807	0.9231	0.9784	0.9698	0.9878	**0.9881**	0.9878
0.2	OA	0.9669	0.9729	0.9360	0.8574	0.9356	0.9808	0.9836	**0.9881**
	AA	0.9597	0.9761	0.8365	0.8297	0.9187	0.9804	0.9803	**0.9866**
	κ	0.9574	0.9705	0.9167	0.8179	0.9169	0.9847	**0.9790**	0.9753
0.3	OA	0.9447	0.9702	0.9241	0.7906	0.8919	0.9789	0.9826	**0.9879**
	AA	0.9322	0.9760	0.8136	0.7699	0.8801	0.9761	**0.9803**	0.9710
	κ	0.9289	0.9694	0.9009	0.7354	0.8997	**0.9845**	0.9777	0.9775
0.4	OA	0.9354	0.9682	0.9200	0.7417	0.8850	0.9773	**0.9790**	0.9781
	AA	0.9167	0.9699	0.8093	0.6887	0.8613	0.9703	**0.9791**	0.9737
	κ	0.9171	0.9678	0.8955	0.6714	0.8567	**0.9837**	0.9678	0.9734
0.5	OA	0.9231	0.9575	0.9189	0.6912	0.8398	**0.9750**	0.9729	0.9711
	AA	0.9135	0.9606	0.8022	0.6945	0.8070	**0.9705**	0.9672	0.9616
	κ	0.9070	0.9652	0.8877	0.6143	0.8153	0.9745	**0.9813**	0.9673

**Table 9:** Reference classes for the *GRSS2013* data set.

No.	Class Name	Train	Test	Labelled Samples
1	Healthy grass	125	1126	1251
2	Stressed grass	125	1129	1254
3	Synthetic grass	70	627	697
4	Trees	124	1120	1244
5	Soil	124	1118	1242
6	Water	33	292	325
7	Residential	127	1141	1268
8	Commercial	124	1120	1244
9	Road	125	1127	1252
10	Highway	123	1104	1227
11	Railway	124	1111	1235
12	Parking Lot 1	123	1110	1233
13	Parking Lot 2	47	422	469
14	Tennis Court	43	385	428
15	Running Track	66	594	660
	Total	1503	13,526	15,029

**Table 10:** Classification results for the *GRSS2013* data set with different classifiers.

ρ	PM	NBC-Spe	NBC-Spa	NBC-LiDAR	KNN	SVM	GGF	R-T	R-LA	R-EU
0	OA	0.6854	0.9668	0.8909	0.6654	0.8463	0.9705	0.8463	0.9717	**0.9733**
	AA	0.6835	0.9568	0.9013	0.6037	0.7993	0.9562	0.8419	0.9599	**0.9628**
	κ	0.6588	0.9640	0.8816	0.6358	0.8328	0.9678	0.8331	0.9692	**0.9708**
0.1	OA	0.6800	0.9602	0.8829	0.6645	0.8068	0.9050	0.8481	0.9654	**0.9697**
	AA	0.6730	0.9495	0.8927	0.6024	0.7583	0.9050	0.8452	0.9536	**0.9608**
	κ	0.6527	0.9598	0.8729	0.6348	0.7898	0.8890	0.8350	0.9640	**0.9687**
0.2	OA	0.6698	0.9524	0.8709	0.6627	0.7600	0.8375	0.8455	0.9533	**0.9597**
	AA	0.6594	0.9387	0.8774	0.6010	0.7126	0.8403	0.8388	0.9414	**0.9423**
	κ	0.6417	0.9483	0.8599	0.6328	0.7392	0.8085	0.8321	0.9492	**0.9537**
0.3	OA	0.6597	0.9406	0.8516	0.6555	0.6923	0.7700	0.8251	0.9342	**0.9520**
	AA	0.6347	0.9269	0.8612	0.5900	0.6367	0.7728	0.8172	0.9215	**0.9359**
	κ	0.6304	0.9355	0.8390	0.6250	0.6658	0.7356	0.8100	0.9286	**0.9419**
0.4	OA	0.6512	0.9274	0.8298	0.6506	0.6249	0.6784	0.8130	0.9113	**0.9329**
	AA	0.6318	0.9172	0.8408	0.5911	0.5855	0.6813	0.8068	0.8994	**0.9231**
	κ	0.6216	0.9213	0.8155	0.6197	0.5929	0.6495	0.7970	0.9037	**0.9275**
0.5	OA	0.6407	0.8890	0.7895	0.6403	0.5435	0.5795	0.7977	0.8799	**0.8939**
	AA	0.6169	0.8841	0.8033	0.5758	0.5117	0.5716	0.7939	0.8736	**0.8873**
	κ	0.6105	0.8796	0.7719	0.6084	0.5055	0.5402	0.7805	0.8697	**0.8832**
